# Effects of Root Extract of Ashwagandha (*Withania somnifera*) on Perception of Recovery and Muscle Strength in Female Athletes

**DOI:** 10.1002/ejsc.12265

**Published:** 2025-02-15

**Authors:** Olivia C. Coope, Andrea Reales Salguero, Tilly Spurr, Andrea Páez Calvente, Aina Domenech Farre, Enrique Jordán Fisas, Beth Lloyd, Julie Gooderick, Maria Abad Sangrà, Blanca Roman‐Viñas

**Affiliations:** ^1^ Blanquerna School of Health Sciences Ramon Llull University Barcelona Spain; ^2^ Seagull Academy Club Esportiu Seagull Barcelona Spain; ^3^ Institute of Sport University of Chichester Chichester UK; ^4^ Department of Physiotherapy ReSport Clinic Barcelona Spain; ^5^ Faculty of Social and Behavioural Sciences Leiden University Leiden Netherlands; ^6^ School of Sport and Exercise Sciences, University of Kent Canterbury UK; ^7^ Faculty of Pharmacy and Food Sciences University of Barcelona Barcelona Spain; ^8^ Faculty of Psychology, Education and Sport Sciences Ramon Llull University Barcelona Spain

**Keywords:** ashwagandha, athlete, female, football, grip strength, recovery, sleep

## Abstract

Ashwagandha is a supplement with the potential to improve exercise performance. However, research on its impact on female athletes remains limited. This study investigates the effects of ashwagandha on exercise recovery and muscle strength in professional female athletes, addressing a gap in understanding its role in this underrepresented population. Female footballers were randomly assigned to a 600 mg/day ashwagandha root extract group (ASH, *n* = 15; age: 26.0 ± 4.9 years, height: 1.66 ± 0.1 m, body mass: 61.5 ± 7.5 kg, and career: 15.2 ± 7.4 years) or a placebo group (PLA, *n* = 15; age: 23.5 ± 5.5 years, height: 1.66 ± 0.1 m, body mass: 61.5 ± 6.0 kg, and career: 13.1 ± 4.9 years). Recovery was assessed with total quality recovery (TQR), Hooper Index (HI) and rate of perceived exertion (RPE). Strength was assessed by hand grip, medicine ball throw (MBT), countermovement jump (CMJ) and peak power. Dietary intake was recorded prior to baseline measurements. Repeated measures ANOVA, Bonferroni test, independent *t*‐tests and ANCOVA were used in the analysis. A significant group × time interaction effect was found for TQR (*p* = 0.026), with the post‐hoc analysis revealing a significant difference between ASH and PLA at 28 days (*p* = 0.039). Perceived sleep quality from HI improved significantly in ASH compared to PLA (*p* = 0.038), with a significant change at 14 days. The ANCOVA analysis highlighted the significant influence of carbohydrate intake on hand grip strength (*p* = 0.005), MBT (*p* < 0.001) and body mass (*p* < 0.001). A dosage of 600 mg of ashwagandha root extract for 28 days may improve TQR and enhance perceived sleep quality in female footballers. Future research should investigate the optimal dosage and test across a broader range of athletic populations.

**Trials Registration:** The trial is registered on ClinicalTrials.gov with the ID NCT06264986

**Summary**

- A 28‐day supplementation with 600 mg of ashwagandha root extract significantly improved total quality recovery in professional female footballers compared to placebo.
- Significant improvements in perceived sleep quality were observed in the ashwagandha group compared to the placebo group, with a marked difference detected at 14 days.
- A 4‐week dosage of 600 mg of ashwagandha root extract appears to be effective, providing context to the optimal dosage length for the supplement.

## 1 Introduction

Muscle strength and overall well‐being are critical determinants of athletic performance (Suchomel, Nimphius, and Stone 2016; Peris‐Delcampo et al. 2024). Female footballers face unique physiological and psychological demands that require specialised interventions to enhance performance and recovery (Randell et al. 2021). Ensuring optimal muscle strength is crucial not only for athletic success but also for injury prevention, overall fitness and longevity in sports. Simultaneously, recovery—which includes factors such as recovery quality, perceived exertion, muscle soreness, stress levels and sleep quality—significantly influences an athlete's ability to maintain consistent high performance (Haller et al. 2022).

In the pursuit of effective interventions to improve performance and well‐being, herbal supplements have gained popularity (Sellami et al. 2018). Among these, *Withania somnifera*, commonly known as ashwagandha, stands out due to its long‐standing use in Ayurvedic medicine and its benefits for stress reduction, enhanced physical performance, cognition and support for recovery (Lopresti et al. 2019; Bonilla et al. 2021; Ziegenfuss et al. 2018; Leonard et al. 2024; Wankhede et al. 2015; Tiwari, Gupta, and Pathak 2021). Despite its growing popularity, research on ashwagandha's impact on muscle strength and recovery, particularly in female athletes, is limited (Bonilla et al. 2021). The optimal dosage to achieve benefits from the supplement remains unclear, with studies employing a wide range of doses and time lengths, from 250 to 1000 mg/day, and durations typically spanning from 8 to 12 weeks. In this randomised controlled trial, a dose of 600 mg/day was selected, as it falls within the proposed safe and effective range and is supported by prior research demonstrating efficacy with comparable doses. Additionally, a duration of 4 weeks aims to evaluate whether the benefits of ashwagandha can be achieved without requiring a longer dosage timeframe. One case study reported potential adverse effects of adrenal hypofunction after ashwagandha supplementation of 858.6 mg/day for 10 weeks (Fry, Fluck, and Han 2022), highlighting the importance of considering individual variability in response to dosage amount and duration. Overall, establishing clear consumption guidelines is essential to maximise the benefits of ashwagandha while minimising potential risks.

A critical aspect of this research is the use of root‐derived ashwagandha extract, as it has negligible levels of a cytotoxic compound, withaferin A, as well as withanone, which is found in leaf extracts of the herb (Priyandoko et al. 2011; Khedgikar et al. 2015). These compounds may induce liver toxicity or adverse reactions with overuse (Siddiqui et al. 2021). Previous reports have documented cases of liver injury linked to ashwagandha supplements that lacked clarity on extraction methods or certification, emphasising the importance of using well‐regulated supplements (Björnsson et al. 2020). The safety and tolerability of root‐extracted ashwagandha are well‐documented in scientific literature, demonstrating normal physiological parameters in both males and females after long‐term use (Verma et al. 2021; Gopukumar et al. 2021).

Improved muscle strength is essential for female athletes, such as footballers who require explosive power and endurance in gameplay (Nobari et al. 2023). Stronger muscles and adequate sleep enhance performance, lower the risk of injury and facilitate quicker recovery (Fleck et al. 1986; Milewski et al. 2014). Despite the potential benefits of ashwagandha, there is a notable gap in the literature regarding its effects on muscle strength and recovery in female athletes (Lopresti et al. 2019). Additionally, existing studies focus on male subjects and often do not report detailed information about dietary intake. This study aims to fill this gap by investigating the effects of 600 mg/day of ashwagandha on perceived recovery and muscle strength in female footballers over a 28‐day period while controlling for dietary intake. The primary outcomes of this study are changes in perceived recovery indicators, with secondary outcomes focusing on muscle strength. It is hypothesised that supplementation will lead to significant improvements in both overall recovery and muscle strength compared to a placebo.

In this study, the aim is to provide novel insights into the potential benefits of supplementation for enhancing performance and recovery in the specific population of female athletes. The findings could have broader implications, informing supplementation practices across various sports where muscle strength, recovery and well‐being are key performance factors. Through a rigorous, controlled supplementation protocol, this study aims to establish a foundation for the safe and effective use of ashwagandha in sports nutrition, ultimately supporting athletes in achieving optimal performance and health.

## 2 Materials and Methods

### 2.1 Study Design

**FIGURE 1:**
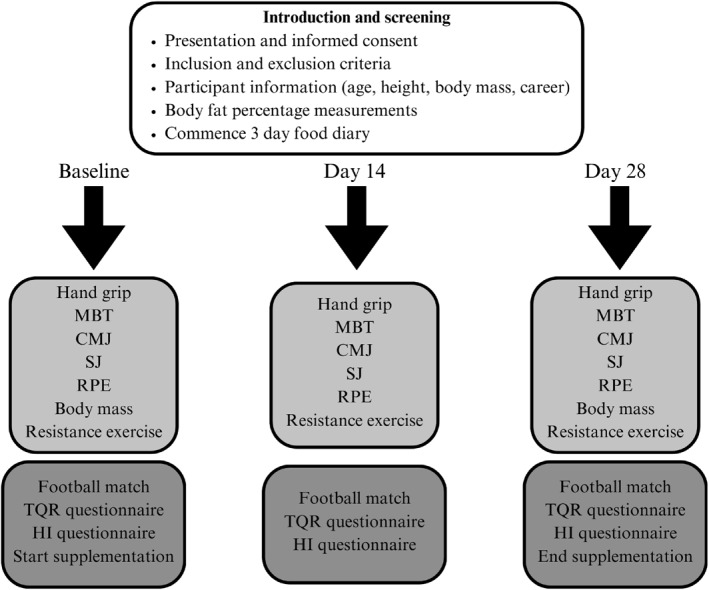
Study timeline. CMJ, countermovement jump; HI, Hooper Index; MBT, medicine ball throw; RPE, rate of perceived exertion; SJ, squat jump; TQR, total quality recovery.

The study employed a randomised, double‐blind, placebo‐controlled design lasting 1 month. A convenience sample was taken from two football clubs. Upon their initial visit, participants were informed about the study and provided written informed consent in accordance with the standards of the Declaration of Helsinki. Figure 1 demonstrates the study timeline. Each participant underwent three measurement sessions to assess muscle strength, followed by the completion of recovery questionnaires the day after scheduled football matches. Data collection included age, body mass, measured using scales (Salter Speedo Mechanical, Kent, United Kingdom), height, measured using a soft tape measure (Wisdompro, Guangdong, China), career duration and medical history. Skinfold measurements were recorded using callipers (Lightstuff Precision Skinfold Calliper, Nevada, United States), following the International Society for the Advancement of Kinanthropometry (ISAK) guidelines. The equations used to estimate the percentage of fat mass were proposed by Carter (Carter and Carter 1982).

Training load was monitored by tracking training volume and duration leading up to each testing session. Baseline muscle strength measurements were conducted during the second visit, which took place in the gyms of the football clubs. Hand grip strength for both the left and right hands was assessed using a dynamometer (CAMRY, California, United States), noted as a reliable tool for evaluating maximum voluntary muscle strength (Huang et al. 2022). Participants underwent a familiarisation session for hand grip prior to the assessment. The right hand was dominant for 28 participants and the left hand for 2 participants, with 1 in each group (ASH and PLA). The test was conducted once for each hand, with participants squeezing once per trial. One assessment per hand was chosen due to its reliability in being comparable to the mean of three assessments (Coldham, Lewis, and Lee 2006). The size of the hand grip was adjusted individually to ensure a proper fit for each participant. Upper body muscle strength and explosiveness were measured using a 5 kg medicine ball (Hypefit, Valencia, Spain) for the medicine ball throw (MBT), shown to be a valid and reliable test for explosive power (Stockbrugger et al. 2001). Participants had a familiarisation session prior to the assessment and were then instructed to throw from a standing position. One assessment of MBT was chosen due to its significant link to hand grip and the reliability of one assessment being comparable to three assessments (Coldham, Lewis, and Lee 2006; Akbar et al. 2022). Each participant threw the ball once, and the distance of the throw was measured using a tape measure. Lower body muscle strength was evaluated through countermovement jump (CMJ) and squat jump (SJ) tests (Markovic et al. 2004). The resulting data from SJ were used in the peak power calculation, in conjunction with body mass (measured in kilogrammes). These measurements were performed using a clinically validated open‐source jump mat (Chronojump Bocosystems, Barcelona, Spain) (Pueo, Penichet‐Tomas, and Jimenez‐Olmedo 2020).

Peak power was calculated using the below mentioned formula (Sayers et al. 1999):

Peakpower(W)=(60.7)×(jumpheight[cm])+45.3×(bodymass[kg])−2055.

Following these assessments, participants engaged in a resistance workout consistent with their regular training regimen in the club gym and evaluated the session's rate of perceived exertion (RPE) using the Borg CR10 scale (Borg 1998) to assess participants' subjective effort and fatigue after the resistance workout. All muscle strength assessments commenced at 7:00 PM at the same site for each visit, under consistent conditions with the same data collection team and a controlled temperature of 20°C. The workout regime was standardised across all participants, except for the order in which equipment was used. The subsequent day, participants completed a football match at 12:00 PM, with the completion of total quality recovery (TQR) and Hooper Index (HI) well‐being visual scale questionnaires at 3:00 PM to assess recovery after the two exercise bouts. TQR covers multiple dimensions of recovery, including nutrition, hydration, sleep, rest and physical activity, assigning a score for each aspect and a valid tool to determine athletes' recovery state after a professional soccer match (Kenttä et al. 1998; Osiecki et al. 2015). HI is a practical and valid assessment tool to determine exercise fatigue in professional soccer by measuring four key parameters: sleep quality, stress levels, fatigue and muscle soreness (Rabbani et al. 2019). Participants rated each parameter on a scale from 1 to 10, with lower scores indicating better conditions (e.g., ‘very, very good’ sleep) and higher scores indicating worse conditions (e.g., ‘very, very bad’ sleep or ‘very, very high’ stress). Menstrual cycles were recorded via self‐report but were not included in the analysis. Recent findings suggest that short‐term hormonal fluctuations have minimal impact on strength or performance, and inconsistent methods of menstrual cycle verification in the literature make it premature to conclude that fluctuations affect recovery or long‐term adaptations (Colenso‐Semple et al. 2023). Furthermore, a study on female endurance athletes found that although the menstrual phase influenced recovery measures, the effect sizes were small, suggesting that the menstrual cycle is unlikely to be a primary determinant of recovery changes but rather one of many potential stressors (De Martin Topranin et al. 2023). Regarding sleep, a study examining the influence of menstrual cycle phase on recovery after high‐ and low‐intensity training found no significant differences in perceived sleep quality or other recovery parameters across menstrual phases (Taylor et al. 2024). Participants subsequently began daily supplementation for 28 days of either 600 mg of root extract of ashwagandha (ASH) with a consistent rate of 5% withanolides or 600 mg of chickpea flour in hydroxypropyl methylcellulose (HPMC) capsules (PLA) and were instructed to take it with water and food. Chickpea flour was selected for its colour similarity to ashwagandha root extract and its gluten‐free properties, in case participants had gluten intolerance. The primary ingredient of the supplement was root extract KSM‐66 ashwagandha (Ixoreal Biomed, Hyderabad, India) supplied by Zenement (BITIO PROJECT SL, Barcelona, Spain). The ingredient is certified by Informed Ingredient, a comprehensive and globally banned substance raw material testing and certification programme for dietary supplements.

Muscle strength tests were repeated on Days 14 (T1) and 28 (T2) following baseline measurements, with corresponding completion of recovery and well‐being questionnaires at these time points. Participants were monitored throughout the duration of the study via club staff members to ensure there were no adverse events. As there would be potential ingestion of a nightshade, it was made clear to report any issues of gastrointestinal discomfort in case of undiagnosed allergy (Kuang et al. 2023; Bashir et al. 2023).

Participants were instructed to record their dietary intake for 3 days, covering one training day, one match day and one recovery day, using the ‘Snap‐N‐Send’ method, which has been validated in a doubly labelled water study and shown to enhance accuracy in assessing total energy intake among athletes (Costello et al. 2019). Participants took photos of all meals and drinks, sending them to a registered sports nutritionist with a brief description of the food items, including portion sizes and food components. There was no limit to the number of messages sent, ensuring comprehensive data collection. Participants were made aware that more accurate reporting will enable a more factual report. This approach attempted to ensure precise recording of dietary habits. The dietary intake was analysed using the Nutritics dietary analysis software (Research edition, v5.096, Dublin, Nutritics, 2019), a reliable tool for quantifying macronutrients and micronutrients, with analysis values positively correlating with plasma markers (Watkins, Freeborn, and Mushtaq 2020). Nutritional parameters assessed included energy intake (kcal), protein (g/day), carbohydrate (g/day), fat (g/day), omega‐3 (g/day) and iron (mg/day), with omega‐3 and iron values estimated rather than precisely measured. Dietary intake is summarised in Table 1.

**TABLE 1:** Nutrient intake and differences by study group.

	ASH (*n* = 15)	PLA (*n* = 15)	*p*‐value
Calories (kcal/kg/day)	21.7 (5.0)	22.28 (4.6)	0.956
Carbohydrates (g/kg/day)	2.31 (0.7)	2.58 (0.6)	0.453
Fat (g/kg/day)	0.8 (0.2)	0.8 (0.2)	0.800
Protein (g/kg/day)	1.23 (0.4)	1.16 (0.3)	0.569
*Iron (mg/kg/day)	0.01 (0.01)	0.01 (0.01)	0.153
*Omega‐3 (g/kg/day)	0.1 (0.03)	0.2 (0.07)	0.832

*Note:* Significance values were calculated using independent *t*‐tests between groups ASH PLA. * indicates estimated values.

Abbreviations: ASH, 600 mg daily dose of ashwagandha root extract; PLA, placebo.

### 2.2 Participants

The study participants (*n* = 30) were between the ages of 18 and 36 years (24.7 ± 5.2 years, 1.66 ± 0.06 m, 61.5 ± 6.6 kg and 14.1 ± 6.2 years in career) and assigned to a professional football club in Barcelona, Spain. Participants in the study were selected based on specific inclusion and exclusion criteria: Eligible participants were female, aged above 18, who were professional footballers playing at a subelite to elite level and who were healthy and free of disease. Exclusion criteria included active supplementation with other ergogenic aids, active pregnancy, use of medications, hormonal contraceptives and allergies to nightshades. Additionally, participants who did not sign the consent form were excluded. The study permitted withdrawal for participants who requested to exit or who failed to complete the required study tests. Participants were randomised using an online randomisation programme (https://www.randomizer.org/). Participant data are found in Table 2.

**TABLE 2:** Participant characteristics and differences by study group.

	ASH (*n* = 15)	PLA (*n* = 15)	*p*‐value
Age (years)	26.0 (4.9)	23.5 (5.5)	0.204
Height (m)	1.66 (0.1)	1.66 (0.1)	0.898
Body mass (kg)	61.5 (7.5)	61.5 (6.0)	0.990
Body mass index (kg/m^2^)	22.2 (1.8)	22.4 (2.6)	0.813
Body fat percentage (%)	25.85 (5.5)	26.75 (5.5)	0.664
Career (years)	15.2 (7.4)	13.1 (4.9)	0.371

*Note:* Significance values were calculated using independent *t*‐tests between groups ASH and PLA.

Abbreviations: ASH, 600 mg daily dose of ashwagandha root extract; PLA, placebo.

### 2.3 Ethical Committee

**FIGURE 2:**
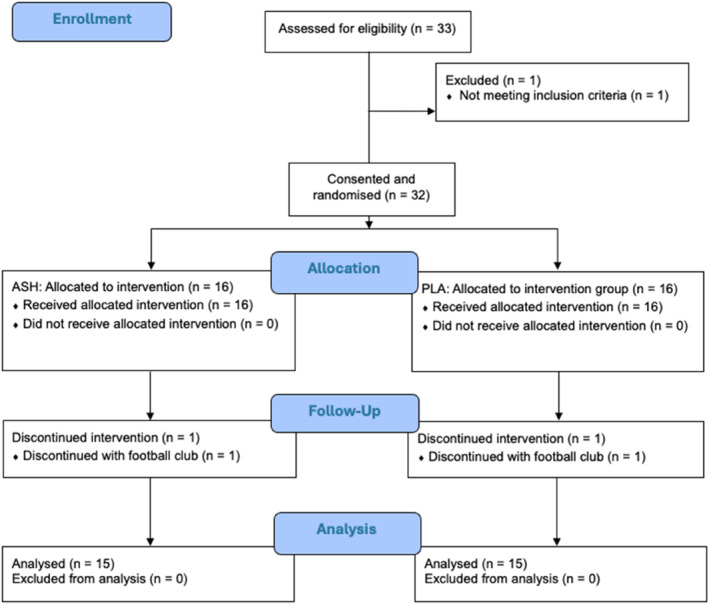
Consolidated Standards of Reporting Trials (CONSORT) diagram. ASH, root extract of ashwagandha; PLA, placebo.

The study and the details of the informed consent form were approved by the Research Ethics Committee of the School of Health Sciences of Blanquerna Institute, University Ramon Llull (CER‐FCSB) on 22 November 2023 (Approval number: 2023‐09‐02). The trial was registered prior to data collection completion on ClinicalTrials.gov with ID NCT06264986. Figure 2 provides a CONSORT (Consolidated Standards of Reporting Trials) flow diagram of the study.

### 2.4 Statistical Analysis

The sample size was generated using G*Power (version 3.1.9.6) and follows guidelines for sample size estimation in sport and exercise science research (Abt et al. 2020). Using the variable TQR in a study analysing ashwagandha and recovery (Tiwari, Gupta, and Pathak 2021), the estimate of the effect size of Cohen's *f* equals 0.67 and is therefore used in the sample size calculation for this proposed research. TQR was chosen as the primary outcome measure of the study, as it provides a comprehensive assessment of recovery in football (Osiecki et al. 2015). Using ANOVA repeated measures between factors, an effect size of 0.67, 80% power and a 5% risk error in a two‐parallel‐group design and three measurements, the minimum sample size is 14 participants with 7 subjects per group. In addition, a further ANCOVA was conducted to assess the impact of dietary intake on strength outcomes, using dietary variables as covariates. This approach allowed for the control of potential confounding effects of diet, providing a clearer understanding of its influence on strength results. All data points were evaluated for normality using the Shapiro–Wilk test. However, as ANOVA is robust for deviations from normality when the sample sizes are small but equal, the analysis proceeds with ANOVA to compare group differences across multiple timepoints (Sullivan, Weinberg, and Keaney 2016). The Bonferroni correction test was used for the post‐hoc analysis, as it efficiently controls Type I errors while making multiple pairwise comparisons (Armstrong 2014). Baseline comparisons between the ASH and PLA groups were made using independent *t*‐tests.

## 3 Results

Significance was set at *p* < 0.05, with data presented as mean ± SD. There were no significant differences between the two groups for the demographic and anthropometric variables (age, height, body mass, body mass index, body fat percentage and career length) at baseline when analysed using independent *t*‐tests, as demonstrated in Table 1. The overall compliance for supplementation during the study was 100%. Compliance was monitored by daily check‐ins with club staff. Adverse events were monitored by the club staff and researchers, asking participants if they had experienced any symptoms related to the supplement. No adverse events occurred in either the ASH or PLA group. Two participants withdrew from the study prior to analysis because of discontinuation from the football team, resulting in 30 participants overall, with 15 in each group. Deviations in normality were found in the data of some of the variables (*p* < 0.05 for MBT, CMJ, peak power and all recovery markers) from the Shapiro–Wilk test results but were assessed with ANOVA given the robustness when the sample size is moderate but equal (*n* = 15 per group) (Sullivan, Weinberg, and Keaney 2016).

### 3.1 Recovery

**FIGURE 3:**
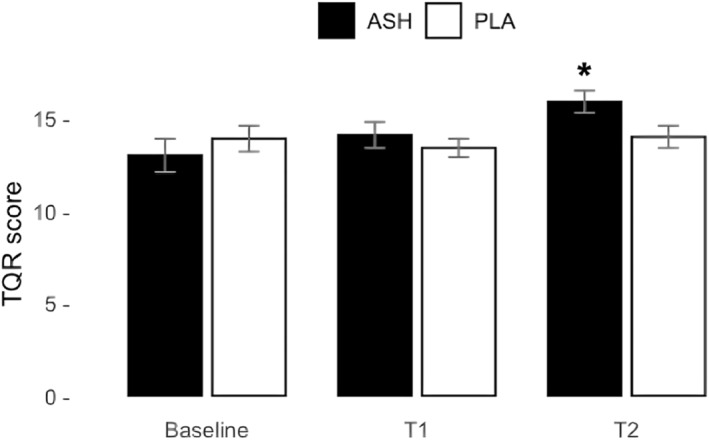
Perception of recovery from total quality recovery (TQR) of ASH (black bars) and PLA (white bars) represented as means ± SD. Significant improvement was found in ASH compared to PLA from ANOVA for the group × time interaction (*p* = 0.026). The post‐hoc Bonferonni analysis revealed a significant difference between ASH and PLA at T2 (*p* = 0.039). Higher scores indicate better conditions. Significant change indicated with *.

**FIGURE 4:**
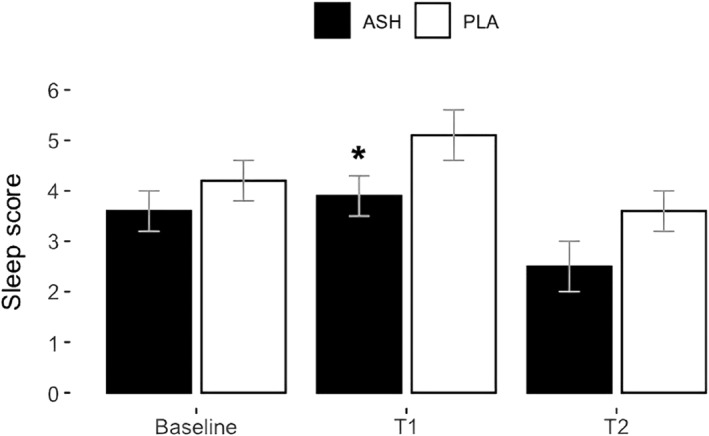
Perception of sleep quality taken from Hooper Index for ASH (black bars) and PLA (white bars) represented as means ± SD. Significant improvement between groups was found in perception of sleep quality determined by ANOVA (*p* = 0.038). The post‐hoc Bonferroni analysis revealed a significant change between ASH and PLA at T1 (*p* = 0.038). Lower scores indicate better conditions on a scale from 1 to 10. Significant change indicated with *.

The recovery scores are detailed in Table 3. ANOVA revealed a significant difference between the group × time interaction for TQR (*p* = 0.026), with scores shown in Figure 3. The post‐hoc analysis of TQR showed a significant difference between ASH and PLA, particularly at T2 (28 days) (*p* = 0.039). The post‐hoc analysis table can be found in Appendix A. Additionally, analysis using ANOVA demonstrated a significant change between groups for perceived sleep quality in ASH compared to PLA (*p* = 0.038), with scores depicted in Figure 4. The post‐hoc analysis revealed significant improvement in perceived sleep quality in ASH compared to PLA, notably at T1 (14 days) (*p* = 0.038). No other significant changes were observed in other assessed recovery parameters.

**TABLE 3:** Effects of the intervention on recovery perception.

		ASH (*n* = 15)	PLA (*n* = 15)	Mean difference	95% CI	Baseline *p*‐value	Group *p*‐value	Group × time *p*‐value
RPE	Baseline	5.8 (0.4)	5.4 (0.6)	0.2 ± 0.4	(5.3, 6.1)	0.567	0.748	0.106
	Day 14	5.4 (0.5)	5.9 (0.6)					
	Day 28	6.1 (0.3)	5.4 (0.5)					
TQR	Baseline	13.0 (0.9)	13.9 (0.7)	0.7 ± 1.2	(13.5, 14.6)	0.404	0.440	0.026*
	Day 14	14.1 (0.7)	13.4 (0.5)					
	Day 28	15.9 (0.6)	14.0 (0.6)					
Sleep	Baseline	3.6 (0.4)	4.2 (0.4)	0.6 ± 1.0	(3.4, 4.2)	0.308	0.038*	0.648
	Day 14	3.9 (0.4)	5.1 (0.5)					
	Day 28	2.5 (0.5)	3.6 (0.4)					
Stress	Baseline	3.7 (0.6)	4.5 (0.6)	3.3 ± 6.9	(3.0, 3.9)	0.406	0.443	0.439
	Day 14	2.7 (0.5)	3.5 (0.6)					
	Day 28	3.2 (0.6)	2.9 (0.3)					
Fatigue	Baseline	4.1 (0.6)	3.6 (0.4)	−1.5 ± 0.9	(3.1, 3.9)	0.518	0.695	0.198
	Day 14	3.2 (0.4)	3.7 (0.5)					
	Day 28	2.9 (0.4)	3.6 (0.5)					
DOMS	Baseline	4.1 (0.5)	3.9 (0.5)	−129.6 ± 75.4	(3.2, 4.0)	0.779	0.969	0.314
	Day 14	4.0 (0.5)	3.7 (0.4)					
	Day 28	2.7 (0.5)	3.3 (0.5)					
HI‐score	Baseline	15.5 (1.9)	16.2 (1.4)	−0.3 ± 1.4	(13.2, 15.5)	0.780	0.308	0.681
	Day 14	13.7 (1.2)	16.0 (1.2)					
	Day 28	11.4 (1.2)	13.5 (1.3)					

*Note:* Baseline *p*‐values were calculated using independent *t*‐tests to compare baseline values between groups. Group *p*‐value and group × time *p*‐value were calculated with ANOVA. Significant change indicated with *.

Abbreviations: 95% CI, 95% confidence interval of the difference between ASH and PLA; ASH, 600 mg daily dose of ashwagandha root extract; DOMS, delayed onset muscle soreness; HI‐score, overall Hooper Index score; Mean difference, difference in change from baseline between ASH and PLA; PLA, placebo; RPE, rate of perceived exertion; TQR, total quality recovery.

### 3.2 Muscle Strength

**FIGURE 5:**
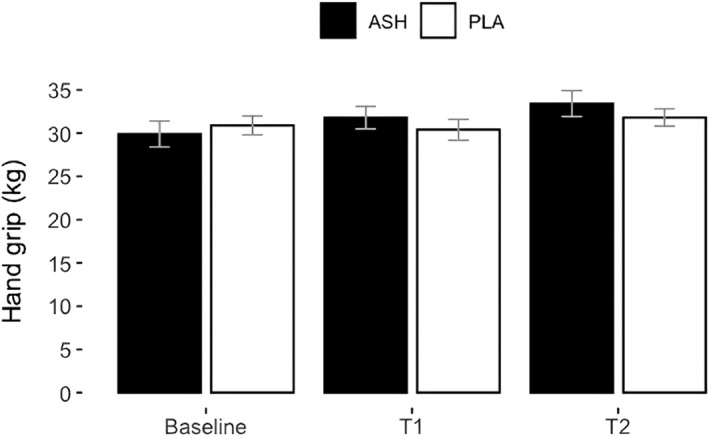
Hand grip strength in kilogrammes for ASH (black bars) and PLA (white bars) represented as means ± SD. A significant group × time interaction was found in ANOVA (*p* = 0.007) indicating that ASH increased hand grip strength compared to PLA; however, the post‐hoc analysis result was not significant.

**FIGURE 6:**
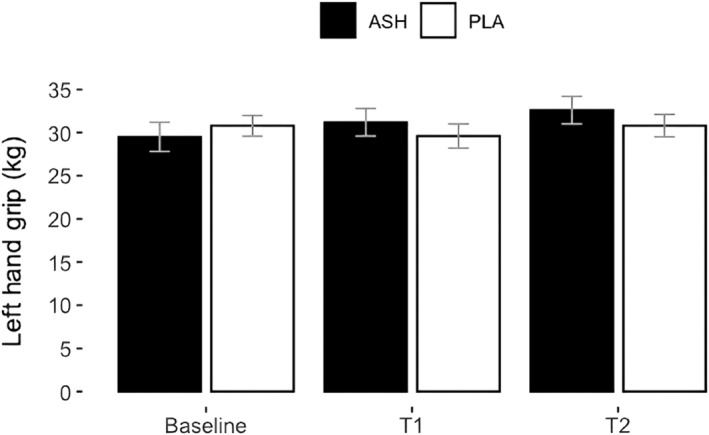
Left‐hand grip strength in kilogrammes for ASH (black bars) and PLA (white bars) represented as means ± SD. A significant group × time interaction in ANOVA (*p* = 0.006) indicates that ASH increased left‐hand grip strength compared to PLA; however, the post‐hoc analysis result was not significant. Among 30 participants, each group had 1 left‐hand dominant participant.

Table 4 summarises the effect of supplementation on muscle strength. The ANOVA analysis revealed a significant group × time interaction effect for changes in right‐hand grip strength (*p* = 0.007) and left‐hand grip strength (*p* = 0.006), shown in Figures 5 and 6, respectively. Among the 30 participants, there were 2 left‐hand dominant participants, belonging to both the ASH and PLA groups. However, the post‐hoc analysis did not determine any significant differences between groups at specific time points. The initial interaction observed in the ANOVA could reflect underlying differences that were not detected in the pairwise comparisons, possibly due to the nature of the data or the adjustments made during post‐hoc testing (Elliott 1996). No other significant changes were found in the muscle strength results.

**TABLE 4:** Effects of the intervention on muscle strength.

		ASH (*n* = 15)	PLA (*n* = 15)	Mean difference	95% CI	Baseline *p*‐value	Group *p*‐value	Group × Time *p*‐value
Hand grip (kg)	Baseline	29.9 (1.5)	30.9 (1.1)	0.6 ± 1.1	(30.3, 32.4)	0.583	0.719	*0.007
	Day 14	31.8 (1.3)	30.4 (1.2)					
	Day 28	33.4 (1.5)	31.8 (1.0)					
Left grip (kg)	Baseline	29.5 (1.7)	30.8 (1.2)	0.7 ± 1.2	(29.6, 31.9)	0.546	0.725	*0.006
	Day 14	31.2 (1.6)	29.6 (1.4)					
	Day 28	32.6 (1.6)	30.8 (1.3)					
Right grip (kg)	Baseline	30.2 (1.4)	31.0 (1.1)	0.6 ± 1.0	(31.0, 33)	0.660	0.739	0.063
	Day 14	32.5 (1.3)	31.2 (1.1)					
	Day 28	34.1 (1.5)	32.9 (0.9)					
MBT (cm)	Baseline	147.0 (10.3)	149.8 (8.7)	3.3 ± 6.9	(147.3, 160.9)	0.837	0.781	0.171
	Day 14	159.4 (7.8)	153.6 (7.7)					
	Day 28	160.7 (8.4)	153.9 (7.9)					
CMJ (cm)	Baseline	25.7 (1.1)	27.2 (0.8)	−1.5 ± 0.9	(26.6, 28.4)	0.276	0.311	0.383
	Day 14	26.7 (1.0)	28.8 (1.0)					
	Day 28	27.9 (1.0)	28.7 (1.5)					
Peak power (W)	Baseline	2238.2 (93.4)	2392.1 (105.2)	−129.6 ± 75.4	(2316.3, 2467.7)	0.283	0.324	0.197
	Day 14	2333.3 (89.7)	2484.6 (89.8)					
	Day 28	2410.0 (82.5)	2492.5 (96.6)					
Body mass (kg)	Baseline	61.5 (1.9)	61.8 (1.5)	−0.3 ± 1.4	(60.3, 63)	0.903	0.907	0.990
	Day 28	61.6 (1.9)	61.8 (1.5)					

*Note:* Baseline *p*‐values were calculated using independent *t*‐tests to compare the baseline values between groups. Group *p*‐value and group × time *p*‐value were calculated with ANOVA. Significant change indicated with *.

Abbreviations: 95% CI, 95% confidence interval of the difference between ASH and PLA; ASH, 600 mg daily dose of ashwagandha root extract; CMJ, countermovement jump; MBT, medicine ball throw; Mean difference, difference in change from baseline between ASH and PLA; PLA, placebo.

### 3.3 Dietary Intake

When nutrient intake covariates were included in the ANCOVA model, carbohydrates, protein, fat and calories demonstrated a significant impact on the muscle strength variables. For hand grip strength specifically, fat (g/kg) (*p* = 0.048) and carbohydrates (g/kg) (*p* = 0.005) had a significant impact. Carbohydrates also demonstrated a significant impact on left‐hand grip strength (*p* = 0.007), right‐hand grip strength (*p* = 0.010), MBT (*p* < 0.001) and body mass (*p* < 0.001). The covariates of omega‐3 (g/day) and iron (mg/day) had no significant impact on the muscle strength variables. The ANCOVA analysis table can be found in Appendix B.

## 4 Discussion

### 4.1 Primary Outcomes

Primary outcomes were established a priori as changes in recovery markers. It was hypothesised that there would be improvements in RPE, TQR, fatigue, DOMS, perception of stress and sleep quality taken from the Hooper Index. Prior research, albeit limited, has consistently shown ashwagandha's potential to improve physiological and psychological well‐being, suggesting beneficial effects on these parameters. Results of this study correlate with previous literature that indicates ashwagandha may improve TQR after exercise (Tiwari, Gupta, and Pathak 2021). Significant improvements in TQR were observed after 28 days of ashwagandha supplementation compared to placebo, with the most pronounced difference occurring at the end of the supplementation period. TQR reflects general measures of exertion and recovery (Brauers et al. 2024), which are critical for sustaining performance and adapting to training demands (Sousa et al. 2024). Female athletes, in particular, face unique recovery challenges due to physiological and hormonal factors that can exacerbate fatigue and impair recovery (van Niekerk, Matzkin, and Christino 2023). A study on semi‐professional female basketball players found a negative relationship between training load and TQR, emphasising the importance of monitoring TQR to optimise recovery and training schedules (Sansone et al. 2018). According to current literature, this is the first study to examine the effects of ashwagandha supplementation on TQR in professional female athletes. The significant findings demonstrate ashwagandha's potential in improving recovery outcomes for female athletes. Significant improvements were noted in the participants' perception of sleep quality following ashwagandha supplementation compared to placebo, with a notable difference at 14 days. Enhanced sleep quality is crucial for athletes (Mah et al. 2018) in terms of supporting recovery, muscle repair and overall physical and mental well‐being (Walsh et al. 2020). In a study focusing on female athletes, it was observed that participating in important matches led to a significant decline in sleep quality, attributed to a notable 354% increase in cortisol levels (O'Donnell et al. 2018). Results of this study concur with previous literature suggesting ashwagandha to be beneficial for sleep quality (Deshpande et al. 2020; Baker et al. 2022). To the authors’ knowledge, no study has presented the benefits of ashwagandha on sleep quality within a female athlete population. This potential ability to enhance sleep quality is promising for athletic cohorts and suggests ashwagandha may have a place within professional sports. Athletes commonly report worse sleep quality than nonathletes (Leeder et al. 2012), with female athletes commonly reporting a worse sleep status than male counterparts (Miles et al. 2022). By promoting better sleep, ashwagandha may contribute to optimised athletic performance, with enhanced sleep factors suggested to be beneficial for sport‐specific skills such as sprint times and jump performance (Mah et al. 2011; Cullen et al. 2019). Furthermore, the ability to enhance sleep quality may have the added benefit of reducing the risk of fatigue‐related injuries and enhancing overall training effectiveness.

The potential mechanism for the improvement in sleep after ashwagandha supplementation may be linked to its modulation of the GABAergic system (Candelario et al. 2015). Ashwagandha has been shown to interact with GABA receptors, which are crucial for regulating sleep, by activating both ionotropic GABA_A_ and GABAρ1 receptors, which help inhibit neuronal activity. Research suggests ashwagandha may enhance GABAρ1 receptor activity more than GABA_A_ receptors, indicating that compounds in ashwagandha may specifically target these receptors to induce relaxation from reduced neuronal activity, resulting in improved sleep quality (Park et al. 2023).

Although previous research suggests that ashwagandha supplementation may improve RPE, perception of fatigue, muscle soreness and stress (Lopresti et al. 2019; Bonilla et al. 2021; Ziegenfuss et al. 2018; Leonard et al. 2024; Wankhede et al. 2015; Tiwari, Gupta, and Pathak 2021), this study did not observe any significant effects. It is possible that the lack of significant findings is due to the relatively short duration of the study, which may not have allowed enough time for ashwagandha to influence these recovery markers. Additionally, variations in individual responses to supplementation and the specific characteristics of the participant population may have contributed to these null results, as noted in previous research on supplementation (Southward et al. 2018). Further research with varied supplementation periods and a crossover design may provide more conclusive evidence on the effects of ashwagandha on these key recovery markers in athletes.

### 4.2 Secondary Outcomes

Based on the current, yet limited, literature on ashwagandha and exercise performance, it was hypothesised that there would be beneficial effects on all markers of strength after a 28‐day supplementation with 600 mg of ashwagandha root extract compared to placebo. For muscle strength, ashwagandha's proposed mechanism is the modulation of cortisol via the hypothalamus‐pituitary‐adrenal (HPA) axis (Lopresti et al. 2019). Cortisol is a hormone known to negatively impact muscle strength and recovery (Katsuhara et al. 2021; Urhausen, Gabriel, and Kindermann 1995). Conversely, exercise, poor sleep and diet affect cortisol levels (Fukuda et al. 2001). Ashwagandha has demonstrated significant improvements in maximum oxygen uptake (VO_2max_) and strength and muscular power of the upper and lower limbs in studies conducted on males and females (Pérez‐Gómez et al. 2020), which may be attributed to the reduction in cortisol. By reducing cortisol levels, ashwagandha may reduce the catabolic effects of stress on muscle tissue, potentially promoting strength gains. The study found a significant improvement in overall hand grip strength (*p* = 0.007) and specifically in left‐hand grip strength (*p* = 0.006) from the initial ANOVA, but the post‐hoc analysis did not deem the results as significant. The study observed overall percentage increases in the ASH group compared to placebo for muscle strength measures of hand grip strength (11.7% vs. 3%), peak power (7.7% vs. 4.2%) and MBT (9.3% vs. 2.7%) from baseline to T2. Despite these findings, rigorous statistical analysis did not reveal significant differences between ASH and PLA for muscle strength.

Hand grip strength is a key indicator of elite athletic performance, correlating with overall body strength, explosive ability, body composition and training experience. It is also related to kinetic chain movements, where it serves as the final point of contact for generating force and is a reliable indicator of major muscle group strength (Cronin et al. 2017; Huebner, Riemann, and Hatchett 2023). For the general population, it is inversely associated with the all‐cause mortality risk (Wang et al. 2022). The assessment of hand grip strength is particularly valuable as it offers a quick, noninvasive method to evaluate an athlete's training progress and physical readiness. Consequently, regular monitoring of hand grip strength can help in tailoring training regimens to optimise athletic performance across various sports disciplines. Although initial analyses indicated improvements in hand grip strength, post‐hoc tests did not confirm these findings as statistically significant, highlighting the need for further research to determine the impact of ashwagandha on muscular performance in athletes.

The significant relationship between carbohydrate intake and muscle strength found in the ANCOVA analysis echoes research that shows carbohydrate supplementation before resistance exercise may help increase the training volume of athletes (Wax et al. 2012). A low carbohydrate intake was noted among participants, corresponding with prior research that monitored fluid and carbohydrate intake in elite female footballers during training and matches (Tarnowski et al. 2022). The low carbohydrate intake may be impairing the athlete's performance, potentially hindering strength gains. This highlights the necessity of considering dietary habits when evaluating physical performance metrics. The role of dietary factors in muscle strength highlights a potential area for future research when analysing supplementation for performance enhancement.

Importantly, no adverse effects were noted during the trial. This suggests that participants likely did not experience gastrointestinal distress or side effects related to adrenal hypofunction induced by ashwagandha, as noted in a previous case study involving a 10‐week supplementation (Fry, Fluck, and Han 2022). Adrenal hypofunction, or adrenal insufficiency, is characterised by insufficient production of cortisol by the adrenal glands (Hahner et al. 2021). Low cortisol levels are often observed in individuals who experience post‐traumatic stress disorder and depression, which can be signalled by anhedonia (Oquendo et al. 2002; Oei et al. 1990). Additionally, anhedonia has been linked to dysfunction of the HPA axis (Fraguas et al. 2015). Because ashwagandha may influence the HPA axis, it is important to avoid excessive ingestion and to use the herbal supplement with caution. This study provides context for dose length recommendations; for athletes or recreational individuals, a duration of 4 weeks may be a safe length to avoid potential interactions with the HPA axis and its function.

### 4.3 Limitations and Strengths

The study has several limitations worth noting. First, there was an absence of clinical testing, such as blood biomarkers, to provide objective physiological data correlating with the observed outcomes. This limits the ability to draw definitive conclusions regarding the biochemical mechanisms underlying ashwagandha's effects on muscle strength and the perception of recovery in the participants. Although sleep quality was measured using self‐reported data, it is recommended that future studies incorporate objective methods to enhance the accuracy of these measurements. Additionally, the study was constrained by a convenience sample, which may affect the generalisability of the findings to broader populations of female football athletes. The additional lack of a crossover design limits the ability to effectively control for possible confounders. Future research employing a crossover design could provide more robust evidence of ashwagandha's effects by minimising inter‐individual variability.

The study has notable strengths, including the monitoring of dietary intake before the study commenced, ensuring consistency in baseline nutritional status across participants; a study analysing the effects of ashwagandha on muscle strength and recovery suggests future studies should attempt to control for dietary intake factors (Ziegenfuss et al. 2018). Furthermore, assessments were conducted at three distinct time points during the supplementation period, allowing a comprehensive evaluation of both short‐term and potentially evolving effects of ashwagandha on muscle strength and perceptive recovery outcomes. An advantage of the study is the use of validated tests to measure recovery and muscle strength. HI and TQR are noninvasive wellness monitoring tools for footballers that can detect early signs of fatigue (Selmi et al. 2022). Hand grip strength is a widely used measure of muscular strength and is associated with athletic performance and recovery—although not a direct measure of football performance, it correlates with overall strength and impulsive ability such as sprinting and jumping (Cronin et al. 2017). Participants were recruited from 2 football clubs, with 1 club providing 20 participants and the other providing 10. A key strength of this study was that both clubs followed equivalent match and training schedules, ensuring that measurements were taken under uniform conditions across both clubs. Another strength of the study is the similar values in participant characteristics across both ASH and PLA groups, as shown in Table 2. Most notably, the study is the first to specifically investigate ashwagandha's effects on female athletes and professional footballers. This focus fills a gap in the current literature, providing novel insights into the potential benefits of ashwagandha supplementation tailored to the unique physiological demands of female athletes in a competitive sporting context. This study used a randomised, double‐blind, placebo‐controlled design, recognised as the gold standard for clinical research.

Future research can explore ashwagandha's effects across different athlete populations, varying supplementation lengths and dosages to elucidate optimal and, above all, safe protocols for enhancing muscle strength, recovery and overall athletic performance. Additionally, investigating the potential interaction between ashwagandha supplementation and specific training regimens could provide valuable insights into personalised sports nutrition strategies for athletes.

## 5 Conclusions

In this double‐blind, placebo‐controlled, randomised study, a significant improvement was observed in TQR and perceived sleep quality among female footballers after a 28‐day supplementation with 600 mg of ashwagandha root extract compared to the placebo group. The study revealed that short‐term administration of ashwagandha can significantly improve recovery markers in female athletes.

Although these findings are encouraging, further studies are necessary to explore the impact of ashwagandha on perceived recovery and muscle strength across varying dosing regimens and extended or reduced supplementation periods. Research should also examine its effectiveness in a wider range of athletes, genders and age groups in maintaining physical strength and general health to explore its potential clinical benefits for individuals seeking to improve physical performance and recovery. It is also recommended that future studies incorporate a crossover design and clinical testing, including cortisol measurements and sleep actigraphy readings, to provide a more comprehensive understanding of ashwagandha's physiological effects.

## Author Contributions

Conceptualisation O.C.C., B.R.‐V., T.S., A.R.‐S., M.A.‐S.; project management O.C.C., B.R.‐V., A.R.‐S.; supplement partnership O.C.C.; data collection O.C.C., A.R.‐S., A.P.‐C., A.D.‐F., E.J.‐F.; data analysis O.C.C., B.L., B.R.‐V.; supervision B.R.‐V., T.S.; writing–original draft O.C.C.; writing–review and editing B.R.‐V., T.S., J.G.; all authors have read and approved the final manuscript.

## Ethics Statement

This study was approved by the Research Ethics Committee of the School of Health Sciences of Blanquerna Institute, University Ramon Llull (CER‐FCSB) on 22 November 2023 (Approval number: 2023‐09‐02). All procedures performed in this study were in accordance with the ethical standards of the institutional research committee and adhered to the Declaration of Helsinki’s ethical standards for conducting human participant research.

## Consent

Informed consent was obtained from all individual participants included in the study. Participation was voluntary, and participants had the right to withdraw at any time without any repercussions. Confidentiality was maintained by anonymising the data and securely storing all information following data protection regulations.

## Conflicts of Interest

The authors declare no conflicts of interest.

## Materials

The supplementation of ashwagandha for this study was generously provided by Zenement (BITIO PROJECT SL). Zenement had no role in the study design, data collection, analysis, interpretation of data or the decision to submit the article for publication.

## Copyright

This manuscript represents original research conducted by the authors and does not infringe on any existing copyrights. All content, data and findings presented are the result of the authors' own study and have not been previously published or disseminated in any form.

## Data Availability

Data and statistical analyses are available upon request on a case‐by‐case basis for noncommercial scientific inquiry and educational use.

## Appendix A Bonferroni Correction Post‐Hoc Analysis of Significant Results

**Table:**

Variable	Group	Comparison	Adjusted *p*‐value
Sleep	ASH	T1 versus baseline	1.000
		T2 versus baseline	0.514
		T2 versus T1	0.124
	PLA	T1 versus baseline	0.538
		T2 versus baseline	1.000
		T2 versus T1	0.082
	Cross‐condition	ASH (baseline) versus PLA (baseline)	0.320
		**ASH (T1) versus PLA (T1)**	**0.038***
		ASH (T2) versus PLA (T2)	0.087
TQR	ASH	T1 versus baseline	1.000
		T2 versus baseline	1.000
		T2 versus T1	0.756
	PLA	T1 versus baseline	0.432
		T2 versus baseline	0.780
		T2 versus T1	1.000
	Cross‐condition	ASH (baseline) versus PLA (baseline)	0.312
		ASH (T1) versus PLA (T1)	0.410
		**ASH (T2) versus PLA (T2)**	**0.039***
Hand grip	ASH	T1 versus baseline	1.000
		T2 versus baseline	0.270
		T2 versus T1	0.851
	PLA	T1 versus baseline	1.000
		T2 versus baseline	1.000
		T2 versus T1	0.906
	Cross‐condition	ASH (baseline) versus PLA (baseline)	0.593
		ASH (T1) versus PLA (T1)	0.431
		ASH (T2) versus PLA (T2)	0.420
Left‐hand grip	ASH	T1 versus baseline	1.000
		T2 versus baseline	0.557
		T2 versus T1	1.000
	PLA	T1 versus baseline	1.000
		T2 versus baseline	1.000
		T2 versus T1	1.000
	Cross‐condition	ASH (baseline) versus PLA (baseline)	0.568
		ASH (T1) versus PLA (T1)	0.452
		ASH (T2) versus PLA (T2)	0.384

## Appendix B ANCOVA Table

**Table:**

Variable		df	SS	Mean square	*F*‐statistic	*p*‐value
Hand grip (kg)	Carbohydrates	1	184.67	184.67	8.30	**0.005**
	Protein	1	0.03	0.03	0.00	0.972
	Fat	1	89.40	89.40	4.02	**0.048**
	Calories	1	10.60	10.60	0.48	0.492
	Omega‐3	1	83.16	83.16	3.74	0.057
	Iron	1	2.67	2.67	0.12	0.730
	Residuals	83	1846.35	22.25		
Left‐hand grip (kg)	Carbohydrates	1	223.17	›223.17	7.69	**0.007**
	Protein	1	7.40	7.40	0.25	0.615
	Fat	1	109.60	109.60	3.78	0.055
	Calories	1	5.57	5.57	0.19	0.663
	Omega‐3	1	97.59	97.59	3.36	0.070
	Iron	1	0.38	0.38	0.01	0.909
	Residuals	83	2409.53	29.03		
Right‐hand grip (kg)	Carbohydrates	1	149.82	149.82	6.99	**0.010**
	Protein	1	5.72	5.72	0.27	0.607
	Fat	1	71.26	71.26	3.33	0.072
	Calories	1	17.24	17.24	0.81	0.372
	Omega‐3	1	69.89	69.89	3.26	0.074
	Iron	1	15.09	15.09	0.70	0.404
	Residuals	83	1777.75	21.42		
MBT (cm)	Carbohydrates	1	14,572.72	14,572.72	17.31	**0.00008**
	Protein	1	1978.60	1978.60	2.35	0.129
	Fat	1	4799.68	4799.68	5.70	**0.019**
	Calories	1	1271.57	1271.57	1.51	0.223
	Omega‐3	1	408.43	408.43	0.49	0.488
	Iron	1	399.00	399.00	0.47	0.493
	Residuals	83	69,881.41	891.94		
CMJ (cm)	Carbohydrates	1	1.47	1.47	0.10	0.757
	Protein	1	32.89	32.89	2.17	0.145
	Fat	1	117.81	117.81	7.76	**0.007**
	Calories	1	123.15	123.15	8.11	**0.006**
	Omega‐3	1	21.01	21.01	1.38	0.243
	Iron	1	58.09	58.09	3.82	0.054
	Residuals	83	1260.85	15.19		
Peak power (W)	Carbohydrates	1	347,179.50	347,179.50	2.95	0.089
	Protein	1	454,034.89	454,034.89	3.86	0.053
	Fat	1	30,657.96	30,657.96	0.26	0.611
	Calories	1	692,904.81	692,904.81	5.89	**0.017**
	Omega‐3	1	340,049.39	340,049.39	2.89	0.093
	Iron	1	454.70	454.70	0.00	0.951
	Residuals	83	9,757,809.06	117,563.96		
Body mass (kg)	Carbohydrates	1	1054.47	1054.47	48.93	**0.0000000006**
	Protein	1	844.79	844.79	39.20	**0.00000002**
	Fat	1	13.14	13.14	0.61	0.437
	Calories	1	34.26	34.26	1.59	0.211
	Omega‐3	1	0.13	0.13	0.01	0.939
	Iron	1	57.44	57.44	2.67	0.106
	Residuals	83	1788.70	21.55		

Abbreviations: CMJ, countermovement jump; df, degrees of freedom; MBT, medicine ball throw; SS, summary of squares.
